# Isolation of indigenous Glutathione producing *Saccharomyces cerevisiae *strains

**Published:** 2016

**Authors:** Tahereh Tahmasebi, Rahim Nosrati, Hamed Zare, Horieh Saderi, Reyhaneh Moradi, Parviz Owlia

**Affiliations:** 1 *Dept. of Microbiology, Faculty of Advanced Science & Technology, Pharmaceutical Science Branch, Islamic Azad University, Tehran, Iran*; 2 *Dept. of Pharmaceutical Biotechnology, School of Pharmacy, Mashhad University of Medical Sciences, Mashhad, Iran*; 3 *Dept. of Pharmaceutical Biotechnology, School of Pharmacy, Shahid Beheshti University of Medical Sciences, Tehran, Iran*; 4 *Molecular Microbiology Research Center, Shahed University, Tehran, Iran.*

**Keywords:** Glutathione, *Saccharomyces cerevisiae*, yeast

## Abstract

**Background:**

Glutathione (GSH) is a non-protein thiol compound, which plays an important role in the response to oxidative stress and nutritional stress. The aim of this study was to isolate indigenous *S. cerevisiae* strains capable of effectively produce GSH.

**Methods:**

One hundred-twenty sweet fruit samples were collected. The strains were isolated on yeast glucose chloramphenicol (YGC) agar medium and identified. The isolates were evaluated for GSH producing on yeast malt (YM) medium. Concentration of glutathione was investigated by recording absorbance of all samples at wavelength 412 nm (Ellman’s method). In addition, optimization of glucose and peptone concentration in culture medium and the effects of various environmental conditions such as temperature (20–35 °C), agitation rate (150–250 rpm), and initial pH (4.0–6.0) were assessed on producing of GSH.

**Results:**

From 120 samples, 80 isolates were identified by morphological, biochemical and molecular tests as *S. cerevisiae*. Five isolates were capable to produce effectively GSH. The optimal culture conditions were agitation rate, 200 rpm; temperature, 30 °C; initial pH, 6; glucose, 30 g/l; and peptone concentration, 5 g/l. In optimal conditions, the amount of derived glutathione was improved compared to YM basal medium and highest GSH concentration (296.8 mg/l) was obtained after cultivation with shaking for 72 h.

**Conclusion:**

The possibility of obtaining *S. cerevisiae* cells with a high GSH intracellular content can be considered an interesting opportunity of furthering the range of application and utilization of this molecule.

## Introduction

Glutathione (GSH) is the most abundant and ubiquitous low-molecular-mass nonprotein thiol widely found in prokaryote to eukaryote organisms (1-3). Structurally, it is a tripeptide composed of L-glutamate, L-cysteine, and glycine (2, 4), and its active group is represented by the thiol (-SH) of a cysteine residue (5, 6). The thiol-reduced (GSH) and disulfide-oxidized (GSSG) are two forms of glutathione (5, 7). The GSH content is more than 98% of total glutathione, and existing in most cells (1, 8). It is synthesized intracellularly in two ATP-dependent and the consecutive steps (1, 6). First, γ-glutamylcysteine synthase (GSH I), catalyzes the formation of γ-glutamylcysteine and in the latter step, glutathione create by the actions of glutathione synthase (GSH II) from combination of γ-glutamylcysteine and glycine (1, 3, 5, 6).

GSH is involved in multiple biological functions in various tissues. Its biological significance is mainly related to the redox and nucleophilic properties (6, 8). GSH plays an essential role in bioreductive reactions, transport processes, enzyme activity and sulfur and nitrogen metabolism (4, 9), protection against harmful oxidative stress and xenobiotic and endogenous toxic metabolite detoxification (5, 10) as well as modulates cell proliferation, apoptosis, immune function, and fibrogenesis (1, 6, 11). Thus, it is a potent, adaptable and a vital self- defense molecule. These characteristics make this molecule as a pharmaceutical compound in therapeutic purpose (3). It is widely used for the treatment of several diseases, such as HIV infections, pancreatic inflammations, liver cirrhosis, and aging (9, 11, 12). In addition, GSH has the potential to be used as a scavenger of toxic compounds (9), food additive and cosmetic industries and sports nutrition (3, 10, 12).

Enzymatic and direct fermentative methods are currently used to produce glutathione on an industrial scale (4, 8). In enzymatic synthesis system, glutathione can be produced using the essential elements, glutathione-generating enzymes (GSH I, GSH II) and its three precursor amino acids in the presence of ATP, Mg^2+^ cofactors and a suitable pH (usually pH 7.5) (4, 8, 9). However, this way is not suited for the successful performance, because the usage of precursor amino acids and require of enzymes to ATP increases the production cost (3). The latter method is an efficient approach to commercially produce glutathione since some yeast strains (3); in particular strains of *Saccharomyces cerevisiae* and *Candida* *utilis* have the ability to accumulate high GSH concentrations in the cells (4, 10). The advantage of the fermentative production of GSH is that can be achieved a high GSH contents by the optimization of fermentation process, using low-cost materials as substrates (8).

The optimum medium and conditions are the basis for high production yield and economy in biological processes (4, 9). Therefore, the optimization of great variation in culture conditions in relation to temperature, pH, agitation and the carbon and nitrogen sources were important in the research of the fermentation process (3). Certain factors have the important effects on the cell growth and lead to an increase in the accumulation of GSH in yeast cells (8).

The aim of this study was to isolate indigenous *S. cerevisiae* strains capable of effectively produce glutathione. The effects of supplementing culture and environmental conditions such as temperature, agitation rate, and initial pH were investigated on GSH production process in detail to maximize GSH intracellular levels in culture of *S. cerevisiae*.

## Materials and Methods

**Yeast Isolation and Identification**

One hundred-twenty sweet fruit samples were collected. One gram of each of the fruit samples was added into 250 ml Erlenmeyer flask containing 90 ml of normal saline followed by preparing serial dilutions. Yeast strains were isolated by surface-streaking onto yeast-extract glucose chloramphenicol (YGC) agar medium and incubated at 30 °C for 2-5 d under aerobic conditions (13). Yeasts were characterized by macroscopic and microscopic morphology and biochemical characteristics, including growth in malt extract, growth at 37 °C, and the ability to hydrolyze urea as well as ferment sugars (glucose, galactose, sucrose, maltose, and lactose) (14, 15). Yeasts were identified according to the criteria of Kurtzman and Fell (15). Molecular identification was performed with PCR using selective primers SC1 (5’-AACGGTGAGAGATTTCTGTGC-3’) and SC2 (5’-AGCTGGCAGTATTCCCACAG-3’) (16). Two selected isolates with the highest GSH producing properties were identified by yeast sequencing of ITS/5.8S rDNA and D1/D2 domain of 26S rDNA. The sequences obtained were compared with those included in the GenBank database using the Basic Local Alignment Search Tool (BLAST at http:// www.ncbi.nlm.nih.gov) (17).

**Glutathione extraction and assay**

GSH production was carried out in 250 ml erlenmeyer flasks containing 100 ml of Malt Extract broth medium (MEB) of the following composition (per liter); malt extract 3 g, soybean peptone 3 g, glucose 20 g, yeast extract 3 g and Sucrose 3 g supplemented with (NH_4_)_2_HPO_4_ 3 g, MgSO_4_ 0.8 g, K_2_HPO_4_ 1 g, KH_2_PO_4_ 1 g. Initial pH was adjusted to 5.8. Each medium were inoculated with ~10^4 ^CFU/ml of a 24h old submerged culture in the MEB, and were incubated at 30°C on a rotary shaker (150 rpm) for 72h. Culture broth obtained at 72h was centrifuged (7000 g for 10 min); collected cells were washed twice with distilled water, suspended in H_2_O, thermally treated at 100 °C for 3 min and then cooled in ice, immediately (9, 10). The obtained suspension was centrifuged and the glutathione concentration in the supernatant was measured by according Ellman’s method (18), by measuring the absorbance of reaction solutions at 412 nm using a spectrophotometer (Jenway 6300, England). A standard curve generated with known amounts of glutathione was used to determine specimen concentrations. Cell growth was determined by measuring the DCW (dry cell weight). DCW was measured after drying the wet cells at 105 °C to constant weight. All of the extraction trials were repeated at least three times.

**Optimization of** **Glutathione production**

Five isolates with the highest intracellular GSH were chosen for further investigations. In a series of preliminary experiments, the effects of different concentrations of the most important components culture medium (glucose and peptone) and various growth conditions on producing of GSH of these isolates were monitored. For determining of optimum concentrations of glucose and peptone components, different concentrations of glucose (10, 15, 20, 30 and 35 g/l) and peptone (3, 4 and 5 g/l) were added to YM medium and intracellular GSH were determined using previously described method. The effects of various environmental conditions such as pH, temperature and shaking were assessed on producing of GSH in the medium that optimize glucose and peptone composition. The effect of temperature was examined by incubation at 25, 30 and 35 °C. For shaking effect, cultures were incubated on a shaker with the different rotation speeds (150, 200 and 250 rpm). Initial pH values of medium ranging from 4 to 6 were adjusted by the addition of either KOH or HCl accordingly.

Data analysis and graph drawing were done using statistics software Graph Pad Prism (v5.0.4) and The Microsoft Excel program.

## Results

**Fig 1:**
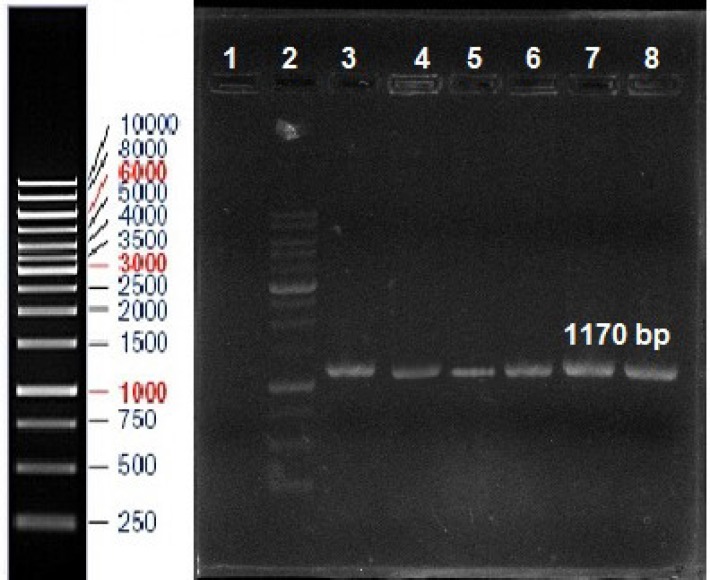
The PCR product of reference *S. cerevisiae* strain and *S. cerevisiae* isolates as follows Lanes 2 and 8, line 1 = negative control, line 2 = 100 bp marker, line 3 = Reference strain *S. cerevisiae*. PTCC 5269, Lane 4 to 8 = Respectively; S2, S3, S4, S34, S52 isolates

 A total of 80 isolates, named S1 to S80, were selected after enrichment, isolation and screening from 120 samples. A total of isolates were identified based on physiological and biochemical tests (15). The isolates were identified by molecular tests with PCR by specific primers. PCR products were about 1170 bp. There was no significant difference in banding pattern compared to the reference strains (Fig. 1). The isolates were identified as *S. cerevisiae. *Two isolates with the highest glutathione production activity, S3 and S34 were chosen for further characterization. Several biochemical and biological indicators as well as sequencing were inspected to figure out the taxonomy of these isolates. The compiled data (not shown) revealed that S3 and S34 isolates must be strains of *S. cerevisiae*. D1/D2 sequences of two isolate (GenBank accession Nos. CBS 8274 and CBS 6414) were all in agreement with the biochemical tests.

Intracellular GSH was measured in all isolates. Rates obtained in samples after 72 h of incubation using only YM broth were in the range of 49 to 234.2 mg/l while cell numbers were adjusted to 10^7^CFU/ml. Biomass concentration in 72 h of incubation ranged from 3.9 to 9 g/l. The five isolates showed the highest intracellular GSH compared to the other isolates. The intracellular GSH result of pure cultures of these strains is shown in Table 1. S3 isolate showed the highest production 234.2 mg/l followed by strain S34 (209.6 mg/l) during 3 days whereas Strain S43 had the lowest glutathione production (49.3 mg/l) during the same period. 

**Table 1:** Intracellular GSH value in the selected isolates with maximum intracellular GSH (S2, S3, S4, S34 and S52) in the YM medium. The isolates were cultivated at 30 °C, pH 5.8 and 150 rpm for 72 h. Values are means of triplicates followed by the standard deviation

Isolates	Biomass (g/l)	Intracellular GSH (mg/L)
**S2**	7± 0.2	179.5 ± 6.9
**S3**	8.6± 0.4	234.2 ± 10.2
**S4**	7.8 ±0.4	182.3 ± 3.7
**S34**	7.3 ±0.8	209.6 ± 9.3
**S52**	7.7 ± 0.5	203.2 ± 4.1

**Fig 2:**
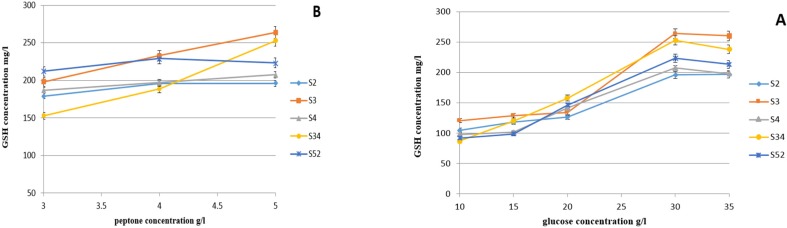
Effect of the addition of different concentration of glucose (panels A) and peptone (panels B) on glutathione production in selected isolates (n=3

Five isolates with the highest intracellular GSH were chosen for further investigations. In a series of preliminary experiments, the analysis of adding different glucose and peptone concentrations in medium demonstrated that intracellular accumulation of GSH was improved by the addition of concentrations of glucose and peptone. The maximum level of GSH production was obtained while 30 g/l glucose was added to the medium. Peptone had a similar impact on intracellular accumulation of GSH. The highest GSH production was observed when peptone concentration reached 5 g/l. Maximum intracellular GSH was obtained when incubation was extending for more than 72 h. The effect of adding different glucose and peptone concentrations on GSH production is shown in Fig. 2.

**Fig 3:**
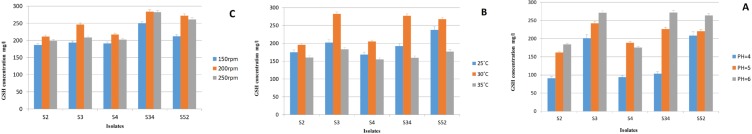
The effect of different initial pH (panel A) incubation temperature (panel B) and agitation rate (panel C) on the intracellular GSH content in the selected isolates (S2, S3, S4, S34 and S52). Yeast strains were grown in modified YM medium. All data points are the means of three replicates. Standard errors are shown by vertical bars

As shown in Fig 3A, increased initial pH value in medium was associated with improved GSH production values. In other words, the intracellular GSH values for all isolates at initial pH 6 were higher than the pH 4 and 5 while a different pattern was observed for S4 strain. The most amount of intracellular GSH content for S4 was detected at initial pH 5. In a comparative experiment set up, maximum intracellular GSH was obtained for S34 at pH 6 (272.3 mg/l) whereas S3 isolate did show the highest intracellular GSH value in pH 5 (242.3 mg/l). The highest intracellular GSH value in samples was obtained 208 mg/l from medium with initial pH 4. The lowest rate was for S4 in pH 4 (93.8 mg/l). 

As shown in Figure 3B, generally, for all selected isolates, the highest GSH content was obtained at temperatures of 30 °C. GSH production of these strains was reduced at 25 °C and 35 °C. In comparison; the lowest levels of glutathione content were observed at 35 °C and the highest production of glutathione were at 30 °C for these isolates. Therefore, the most GSH production was obtained for S3 after 72 h at 30 °C (282.3 mg/l). Although, under same conditions, the maximum levels of glutathione content were 237.7 mg/l and 183.1 mg/l at 25 and 35 °C, respectively. 

The results of different rotation speeds effect on GSH production showed that the highest amount of glutathione was evidenced at agitation rate of 200 rpm whereas the lowest levels of glutathione were detected in the rotation speed of 150 rpm. This was gradually decreased when incubation was extended beyond 250 rpm. Therefore, GSH production of selected isolates was reduced at both high and low rotation speeds of 200 rpm. S34 isolate was capable of the highest intracellular GSH at agitation rates 150, 200 and 250 rpm. The effect of rotation speeds on GSH production is shown in Fig. 3C.

**Fig 4:**
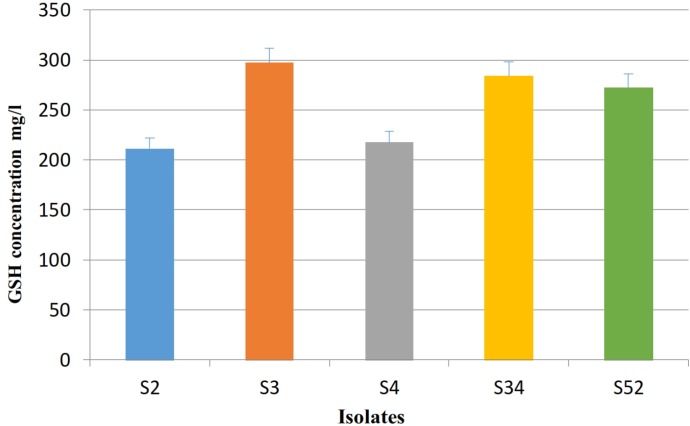
Intracellular GSH value in the selected isolates with maximum intracellular GSH (S2, S3, S4, S34 and S52) in the optimal medium. The isolates were cultivated at 30 °C, pH 6

The optimal culture conditions were agitation rate, 200 rpm; temperature, 30 °C; initial pH, 6; glucose, 30 g/l; and peptone concentration, 5g/l. In optimal conditions, the amount of derived GSH was improved compared to YM basal medium and highest glutathione concentration (296.8 mg/l) was obtained after cultivation with shaking for 72 h (Fig. 4).

**Table 2:** Concentration of GSH reached by other authors in different conditions of culture

Strain	Glucose (%)	Peptone (g/l)	T (°C)	Agitation (rpm)	pH	GSH (mg/l)	Reference
*S. cerevisiae* FF-8	3	5	30	100	6	204	(20)
*S. cerevisiae* T65	3.2		30	180	-	153.2	(21)
*S. cerevisiae* WSH-J701	3	9	10	200	5	64.7	(22)
*S. cerevisiae* CBS 1171	2% beet molasses	soybean peptone	24	200	5.8	81.1	(9)
*S. cerevisiae* ATCC 7754	6.27	-	30	150	-	124.9	(23)
*S. cerevisiae* T65	3.2	-	30	180	5.5	329.3	(19)
*S. cerevisiae* T65	7	5	30	180	-	74.6	(4)
*S. cerevisiae* CBS 1171	2% beet molasses	soybean peptone	28	200	5.8	290	(10)
*S. cerevisiae S-8H*	2.5	4	30		6	160	(28)
*S. cerevisiae*	5.4	5	20	300	5	154.5	(3)

## Discussion

Although GSH is wildly distributed in the nature, the extraction of this tripeptide from *S. cerevisiae* and *C. utilis* yeasts seems to be the only commercial existing biotechnological production method to date (8). “The higher intracellular GSH content, the more products could be obtained supposing the biomass concentration keeps constant” (4). Therefore, most industrial microbiologists put emphases on the optimization of fermentation process to increase the intracellular GSH content of yeast; however, GSH contents of the wild-type strains are usually variable (0.1–1% dw) (8). This has lead researchers to seek out isolation of GSH production yeasts from nations and to explore methods for screening these strains and subsequent evaluations for specific criteria. Nevertheless, the performance of GSH production yeasts is severely influenced by culture conditions and environmental conditions of the targeted regions (3, 19). 

Many studies have tried to improve the GSH production by supplementing certain materials, such as glucose, minerals etc. Cha et al. (20) assessed the influence of carbon and nitrogen sources on glutathione production by *S. cerevisiae* FF-8, and the glutathione concentration achieved using this medium increased to 204 mg/l compared to YM basal medium. The effects of amino acids on GSH production were investigated by Wen et al. (21), and the cell biomass and GSH yield were 9.4 g/L and 153.2 mg/L, respectively. Wei et al. (22) studied the effect of surfactants on extracellular accumulation of GSH and obtained a concentration of 64.7 mg/l. The possibility to obtain *S. cerevisiae* cells with a high intracellular GSH content (81.1 mg/l) (9). Liu et al. (23) studied the medium optimization and found that glucose, peptone, and magnesium sulfate were suitable components for cell growth and GSH production (124.9 mg/l) by *S. cerevisiae* ATCC 7754. Zhang et al. (4) optimized the medium composition for GSH production in shake-flask and in the optimal point achieved 74.6 mg/l. 

In this study we investigated the potential of GSH production by *S. cerevisiae* and interested to study the culture conditions to improve the GSH production in these strains. Similar to previous studies (24, 25), the results demonstrated that intracellular GSH content was improved by the addition of concentrations of glucose and peptone. An increase in GSH accumulation was observed with increase in glucose supply up to 30 g/l; however, high glucose concentration seemed to have an inhibitory effect. The reason might be GSH biosynthesis is a high-energy consumption process and ATP precursor is required in the synthesis of peptide bonds of GSH (24, 25). The ATP required is derived from the metabolism of glucose via the glycolysis and tricarboxylic acid pathways and the respiration chain (25).The increasing concentration of glucose to 30 g/l supply sufficient ATP for GSH biosynthesis. With an increase of glucose concentration, the respiration–fermentation burden was also aggravated and this would enhance the production of reactive oxygen radicals (18). Moreover, additional capacity of uptake glucose by yeast cells is used for other metabolic pathway (26, 27). At high oxidative state, two molecules of reduced GSH formed one molecule of oxidized GSH (24).

This study demonstrated that the GSH concentration was also affected by the level of temperature, rotation speeds and pH. At the high level of agitation rate, the strains produce a greater GSH concentration than at low levels due to an improvement in oxygen supply, which would be beneficial for ATP production (24, 25). As discussed above, biosynthesis of GSH are high-energy consumption processes. Thus, under aerobic conditions, GSH accumulation will occur favorably because the abundant supply of precursors ATP formed in glucose metabolism, which is supplemented in the medium. However, if the aeration rate highly improves, this might cause ATP shortage and the synthesis of GSH will be alleviated (26). In addition, in high rotation speed, without supplementing three amino acid precursors in the medium, the intracellular concentrations of the three amino acids will be maintained at physiological levels and GSH synthesis will be subjected to product inhibition (19, 24).

The intracellular GSH concentration was associated with cell biomass (10). Generally, under favorable environmental conditions, the growth range of strains increase and consequently could function as efficient metabolic activities. While, under unfavorable conditions, the maximum energy of cell use for adaptation of various conditions and a noticeable reduction of biosynthesis was observed; although, these isolates would functionally be active.

This study demonstrated that in optimal conditions, the amount of derived glutathione was improved compared to YM basal medium and highest content of GSH in cells (296.8 mg/l) which was higher than the concentration obtained by many studies (3, 4, 9, 10). The concentrations of GSH observed by other authors in different conditions of culture are presented in Table 2.

## Conclusion

The optimal culture conditions were agitation rate, 200 rpm; temperature, 30 °C; initial pH, 6; glucose, 30 g/l; and peptone concentration, 5 g/l. In optimal conditions, the amount of derived glutathione was improved compared to YM basal medium and highest glutathione concentration (296.8 mg/l). The possibility of obtaining *S. cerevisiae* cells with a high GSH intracellular content can be considered an interesting opportunity of furthering the range of application and utilization of this molecule.
